# Achieving Intrinsically Self‐Healing Fabrics with Breathability, Surface Self‐Repairing, and Underwater Adhesion via Nanoparticle‐Polymer Gel Synergistic Coatings

**DOI:** 10.1002/smll.202505120

**Published:** 2025-06-18

**Authors:** Tsung‐Hung Tsai, Lin‐Ruei Lee, Yi‐Chun Fan, Ruo‐Yun Wei, Chia‐Wei Chang, Huan‐Wei Lin, Yu‐Chun Lin, Tse‐Yu Lo, Kesavan Manibalan, Ji Lin, Yen‐Shen Hsu, Yu‐Hsuan Tseng, Hsun‐Hao Hsu, Jiun‐Tai Chen

**Affiliations:** ^1^ Department of Applied Chemistry National Yang Ming Chiao Tung University Hsinchu 300093 Taiwan; ^2^ Center for Emergent Functional Matter Science National Yang Ming Chiao Tung University Hsinchu 300093 Taiwan

**Keywords:** dip‐coating, intrinsic self‐healing, polymeric ionic gel, surface self‐repairing, underwater healable

## Abstract

Self‐healing fabrics have attracted increasing attention as a sustainable solution to extend fabric lifespan and reduce material waste. However, most reported self‐healing fabrics rely on extrinsic systems with limited healing cycles, or intrinsic polymers that compromise breathability due to irreversible layer adhesion. In this work, a simple and scalable dip‐coating method is reported to fabricate intrinsically self‐healing fabrics that retain their porous structure and air permeability. Commercial fabrics are coated with surface‐modified silica nanoparticles (SiNPs) and a polymeric ionic gel (PIG) composed of poly(vinylidene fluoride‐co‐hexafluoropropylene) (PVDF‐HFP) and ionic liquid ([EMI]^+^[TFSI]^−^). The SiNPs enhance coating durability and hydrophobicity, while the PIG enables intrinsic self‐healing via ion–dipole interactions. The resulting fabrics exhibit rapid and repeatable self‐healing at room temperature, even underwater, while maintaining breathability and repellency to various liquids. Furthermore, the PIG solution can be applied as a reversible adhesive or touch fastener, highlighting its multifunctionality. This study provides a versatile platform for the development of smart textiles with enhanced durability, self‐healing, and customizable adhesion properties.

## Graphical abstract

This study reports a simple and scalable dip‐coating method to fabricate intrinsically self‐healing fabrics with retained porous structure and air permeability. Commercial fabrics are coated with surface‐modified silica nanoparticles (SiNPs) and a polymeric ionic gel (PIG) composed of poly(vinylidene fluoride‐co‐hexafluoropropylene) and ionic liquid ([EMI]^+^[TFSI]^−^). The SiNPs enhance coating durability and hydrophobicity, while the PIG enables intrinsic self‐healing via ion–dipole interactions.

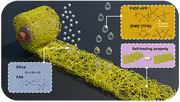

## 1 Introduction

Fabrics are essential materials in daily life, widely used in clothing, footwear, and tents, offering comfort, durability, and aesthetic appeal.^[^ ^1^ ^]^ However, regardless of care, fabrics inevitably become torn, cut, or worn over time, requiring maintenance or replacement. This situation not only causes inconvenience but also increases costs. Moreover, the rise of functional fabrics has amplified recycling and degradation difficulties, contributing to resource waste and environmental pollution.^[^ ^2^, ^3^ ^]^ As of 2017, global plastic production reached 9.2 billion metric tons, but only 6.5% was recycled, with the remainder becoming waste.^[^ ^4^ ^]^

To address these issues, self‐healing fabrics have emerged as a promising solution.^[^ ^5^, ^6^, ^7^ ^]^ These polymer‐based materials can autonomously repair their structure and functionality after damage, reducing the impact of tears and cuts and extending the fabric lifespan. Self‐healing polymers are generally classified as extrinsic or intrinsic.^[^ ^8^, ^9^ ^]^ Extrinsic self‐healing polymers contain pre‐added healing agents encapsulated with catalysts; upon damage, these agents release precursors into cracks to initiate healing reactions.^[^ ^10^, ^11^ ^]^ However, extrinsic systems have limitations, including finite healing cycles and complex compositions. In contrast, intrinsic self‐healing polymers rely on dynamic bonds such as hydrogen bonds,^[^ ^12^, ^13^ ^]^ host‐guest interactions,^[^ ^14^, ^15^ ^]^ ionic interactions,^[^ ^16^, ^17^ ^]^ and ion‐dipole interactions.^[^ ^18^, ^19^ ^]^ These materials do not require additional healing agents, allow multiple healing cycles at the same damage site, and offer more reliable self‐healing capabilities, broadening their potential applications.^[^ ^20^, ^21^ ^]^

Despite intrinsic self‐healing polymers having superior healing properties, most self‐healing fabrics use extrinsic systems because intrinsic polymers often lose their porous structure, causing the fabric layers to adhere irreversibly.^[^ ^22^, ^23^ ^]^ In our previous research,^[^ ^24^, ^25^, ^26^ ^]^ intrinsic self‐healing fabrics were successfully fabricated via electrospinning by blending self‐healing polymers with crystalline polymers or elastomers, avoiding structural collapse. However, electrospinning presents challenges including limited scalability, long processing times, and insufficient durability.

To overcome these limitations, this study introduces a simple and scalable method for preparing intrinsic self‐healing fabrics through dip‐coating. Commercial fabrics (rayon) are coated with modified silica nanoparticles (SiNPs) and polymeric ionic gel (PIG) solutions composed of poly(vinylidene fluoride‐co‐hexafluoropropylene) (PVDF‐HFP) and ionic liquid ([EMI]^+^ [TFSI]^−^). Dip‐coating is advantageous due to its low cost,^[^ ^27^ ^]^ electricity‐free operation, room‐temperature processing,^[^ ^28^ ^]^ and minimal waste generation.^[^ ^29^ ^]^ Incorporating SiNPs enhances fabric durability,^[^ ^30^ ^]^ while the PIG layer provides intrinsic self‐healing capability via ion‐dipole interactions.^[^ ^31^ ^]^

In this work, intrinsic self‐healing fabrics demonstrate room‐temperature healing capability, even underwater, and retain breathability and hydrophobicity, significantly reducing production costs and processing times. Moreover, the developed dip‐coating solution shows potential applications as adhesives and touch fasteners. Unlike previous studies that mainly focused on surface functional restoration,^[^ ^30^, ^32^, ^33^, ^34^ ^]^ our approach enables actual structural self‐healing of the fabric. This method represents a simple, scalable advancement with significant promise for smart textile applications.

## 2 Results and Discussion

**Figure 1:**
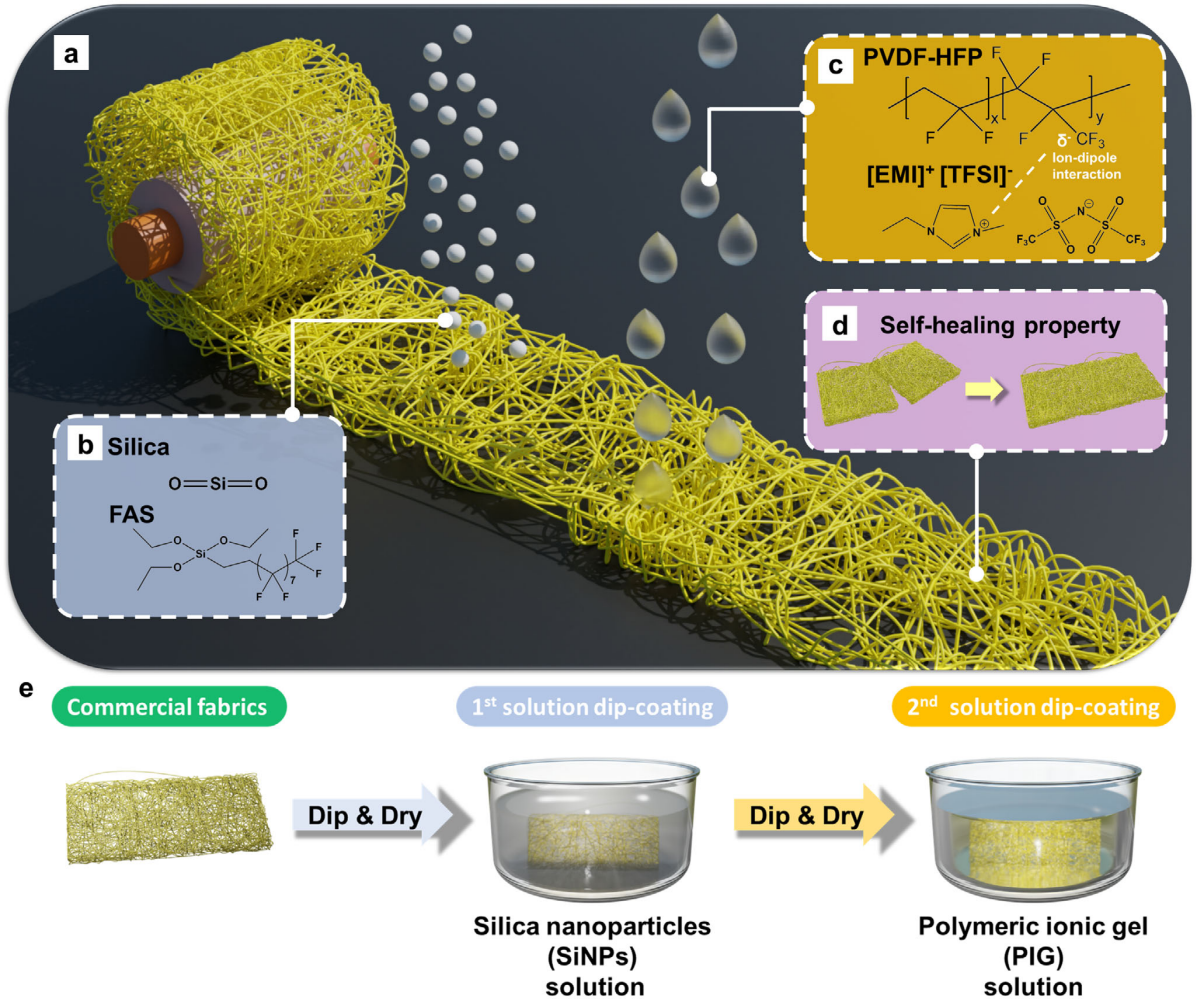
a) Conceptual illustration of the self‐healing fabrics. b) Chemical structures of silica nanoparticles and 1H,1H,2H,2H‐perfluorodecyltriethoxysilane (FAS). c) Chemical structures and ion‐dipole interactions between polymer (PVDF‐HFP) chains and ionic liquids ([EMI]^+^ [TFSI]^−^). d) Schematic illustration of the self‐healing property. e) Experimental process of the 1st and 2nd solution dip‐coating steps for fabricating the self‐healing fabrics.

**Figure** **1**a presents a conceptual illustration of the intrinsically self‐healing fabrics. This approach involves coating commercial fabrics with two essential components: SiNPs and a PIG solution. Through a two‐step solution dip‐coating process, fabrics with intrinsic self‐healing properties are successfully fabricated. The chemical structure of the SiNPs, modified with 1H,1H,2H,2H‐perfluorodecyltriethoxysilane (FAS), is shown in Figure 1b. This modification significantly enhances the adhesion between the SiNPs and the subsequently applied PIG layer, primarily due to the presence of fluorine in FAS. At the same time, fluorinated groups on SiNPs enhance the interfacial compatibility between the PIG and fabric substrates by lowering surface energy and promoting physical anchoring at the fiber‐gel interface. This interfacial enhancement contributes to the overall durability of the coating. Moreover, the modified SiNPs exhibit increased hydrophobicity; even when the PIG layer is degraded or removed, the fabrics can still retain their water‐repellent properties.

Figure 1c illustrates the chemical structure of the PIG, which is composed of poly(vinylidene fluoride‐co‐hexafluoropropylene) (PVDF‐HFP) and the ionic liquid 1‐ethyl‐3‐methylimidazolium bis(trifluoromethylsulfonyl)imide ([EMI]^+^ [TFSI]^−^, abbreviated as EMITFSI). PVDF‐HFP is a copolymer consisting of two monomeric units: vinylidene fluoride (VDF) and hexafluoropropylene (HFP), each imparting distinct physical characteristics. VDF contributes to higher crystallinity and lower polarity, whereas HFP provides lower crystallinity and higher polarity. By tuning the VDF‐to‐HFP ratio, the resulting PVDF‐HFP copolymer can be tailored to exhibit either of these opposing characteristics. Due to the strong polarity of HFP, partial negative charges are distributed over the ─CF₃ functional groups. Upon the incorporation of EMITFSI, the [EMI]^+^ cations interact with the ─CF₃ groups through ion–dipole interactions, acting as physical crosslinkers between polymer chains, as illustrated in Figure S1 (Supporting Information). These interactions give rise to dynamic bonding within the gel network, thereby imparting the intrinsic self‐healing capability to the material. In this study, two types of PVDF‐HFP are utilized: amorphous PVDF‐HFP (a‐PVDF‐HFP) with 45 mol% HFP and crystalline PVDF‐HFP (c‐PVDF‐HFP) with 5 mol% HFP. The use of these two variants enables the systematic investigation of the resulting self‐healing behavior and mechanical properties, thereby offering tunability for specific functional applications. Figure S2 and Video S1 (Supporting Information) contain photos and a video for demonstrating the self‐healing property of the PIG by creating a notch and dropping water. After healing, the notched area of the PIG has been recovered. As a result, no water can go through the PIG and the PIG can still be stretchable. The SEM image (Figure S3, Supporting Information) of the notch area also confirmed the healing capability with a small protrusion ≈10 µm.

The self‐healing capability of the coated fabrics is demonstrated in Figure 1d. Even after being cut, the fabrics are able to restore their structure under applied pressure, clearly exhibiting intrinsic healing behavior. The fabrication process of these self‐healing fabrics is illustrated in Figure 1e. Commercial rayon fabric is chosen due to its wide availability and excellent thermal stability. The dip‐coating process begins with immersion of the fabric into a solution of SiNPs, forming the initial coating layer. After drying at room temperature for 10 min, the fabric undergoes a second immersion in the PIG solution, completing the two‐step coating procedure. This straightforward yet effective method ensures a uniform coating and imparts reliable surface self‐repairing functionality to the fabrics.

**Figure 2:**
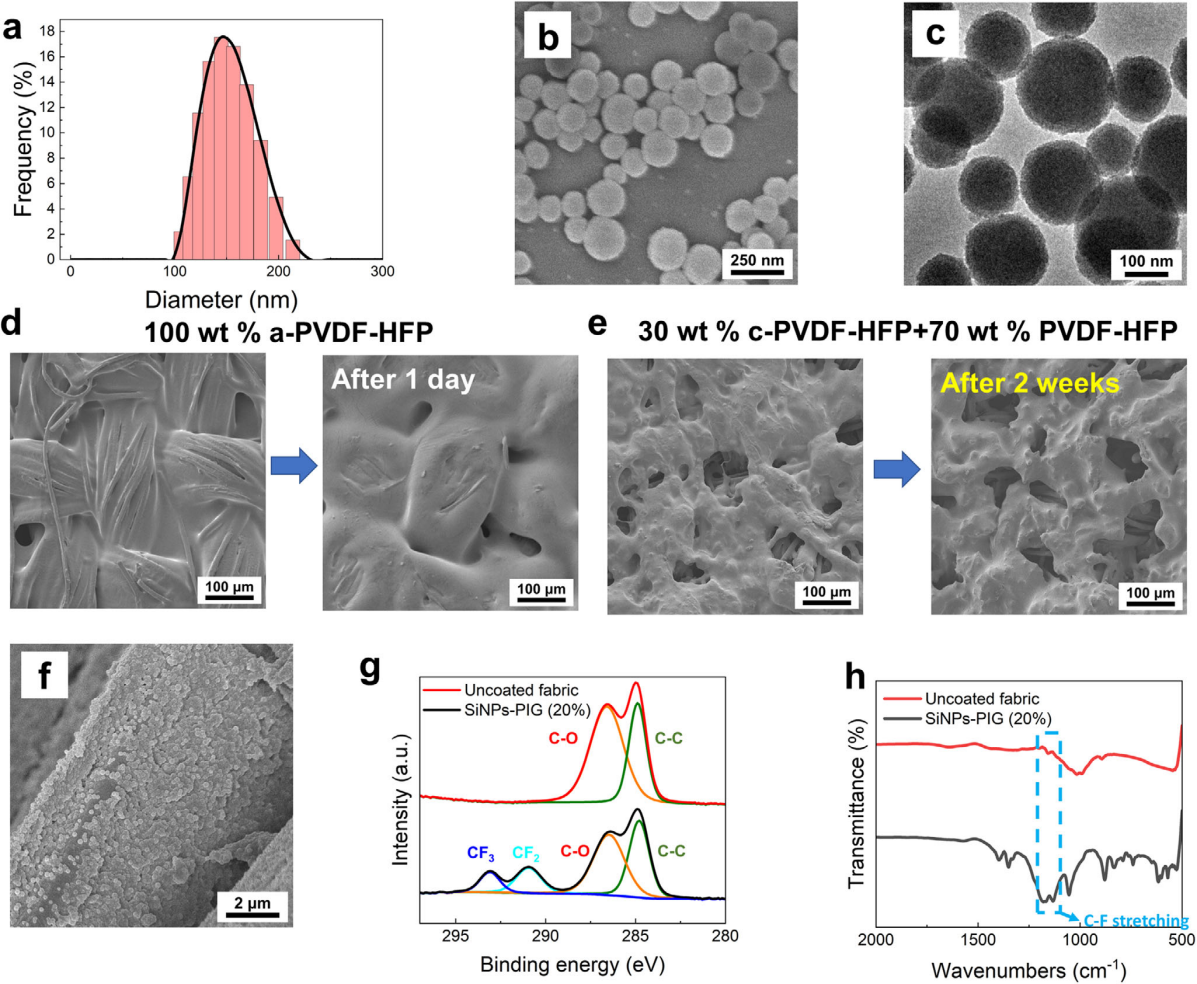
Characterizations of the SiNPs and coated fabrics. a) DLS spectrum of the SiNPs. b) SEM image of the SiNPs. c) TEM image of the SiNPs. d) SEM images of the coated fabrics (100 wt.% a‐PVDF‐HFP) right after coating and after one day. e) SEM images of the coated fabrics (30 wt.% c‐PVDF‐HFP+70 wt.% PVDF‐HFP) right after coating and after two weeks. f) SEM image of the fabrics coated with SiNPs and 10 wt.% PIG solutions. g,h) XPS C1s spectra and FTIR spectra of the uncoated fabrics and fabrics coated with SiNPs and 20 wt.% PIG solutions.

The SiNPs are synthesized using tetraethyl orthosilicate (TEOS), as described in the experimental section. Dynamic light scattering (DLS) analysis is performed to determine the size distribution of the SiNPs, which are diluted tenfold with ethanol before measurement, as shown in **Figure** **2**a. The particle diameters are estimated to be ≈100 to 200 nm, confirming a relatively uniform size distribution. Further morphological characterization is conducted using scanning electron microscopy (SEM) and transmission electron microscopy (TEM), as shown in Figure 2b,c, respectively. These images reveal that the SiNPs exhibit a spherical morphology, consistent with the expected structural characteristics of the synthesized nanoparticles.

After the two‐step dip‐coating process, the intrinsically self‐healing fabrics are obtained. In this study, the polymer component of the PIG solution is prepared using blends of a‐PVDF‐HFP (45 mol% HFP) and c‐PVDF‐HFP (5 mol% HFP) at various blending ratios. SEM images of fabrics coated using only a‐PVDF‐HFP immediately after preparation and after one day are shown in Figure 2d. The fused surface morphology observed in the SEM images arises from the low glass transition temperature (*T*_g_) and high segmental mobility of a‐PVDF‐HFP, which promotes gradual film fusion over time. This process fills the voids in the fabric structure and leads to a noticeable reduction in breathability after one day. These observations are consistent with previous work.^[^ ^24^ ^]^ To address this issue, the blending ratio of a‐PVDF‐HFP to c‐PVDF‐HFP is adjusted from 1:0 to 7:3, thereby increasing the polymer crystallinity. Figure 2e presents SEM images of fabrics coated with the 7:3 a‐PVDF‐HFP to c‐PVDF‐HFP ratio (30 wt.% c‐PVDF‐HFP + 70 wt.% PVDF‐HFP) immediately after coating and after two weeks. The results demonstrate that the incorporation of c‐PVDF‐HFP enhances the structural stability of the coating, maintaining fabric morphology even after extended periods, while also preserving breathability at room temperature. To further examine SiNP attachment on fabric surfaces, a lower PIG concentration (10 wt.%) is used, as shown in Figure 2f. The SEM image indicates that the SiNPs are densely adhered to the fabric surface, aided by the adhesive nature of the PIG layer. The thermogravimetric analysis (TGA) results of a‐PVDF‐HFP, EMITFSI, and PIG are shown in Figure S4 (Supporting Information). The decomposition temperature of a‐PVDF‐HFP, EMITFSI, and PIG are ≈454.5, 407.3, and 399.4 °C, respectively, indicating good thermal stabilities of the materials.

X‐ray photoelectron spectroscopy (XPS) is employed to confirm the presence of coatings on the fabrics. Figure 2g displays the C_1s_ XPS spectra for uncoated fabrics and those coated with SiNPs and 20 wt.% PIG solution. Compared to the uncoated sample, the coated fabrics exhibit distinct CF₃ and CF₂ peaks, originating from PVDF‐HFP and EMITFSI in the PIG layer. Furthermore, the full XPS spectra (Figure S5, Supporting Information) show a significant increase in F_1s_ and N_1s_ signals, further confirming the successful deposition of the PIG. It is noteworthy that under these conditions (20 wt.% PIG), the SiNPs are fully embedded within the PIG, making the Si signal undetectable. Fourier‐transform infrared (FTIR) spectroscopy is also conducted (Figure 2h). In contrast to the uncoated fabrics, the coated samples exhibit characteristic C–F stretching and CF₂ antisymmetric stretching peaks at 874 and 1180 cm^−^¹, respectively, corresponding to PVDF‐HFP and EMITFSI in the PIG layer. These results collectively confirm the successful deposition of the PIG coating.

**Figure 3:**
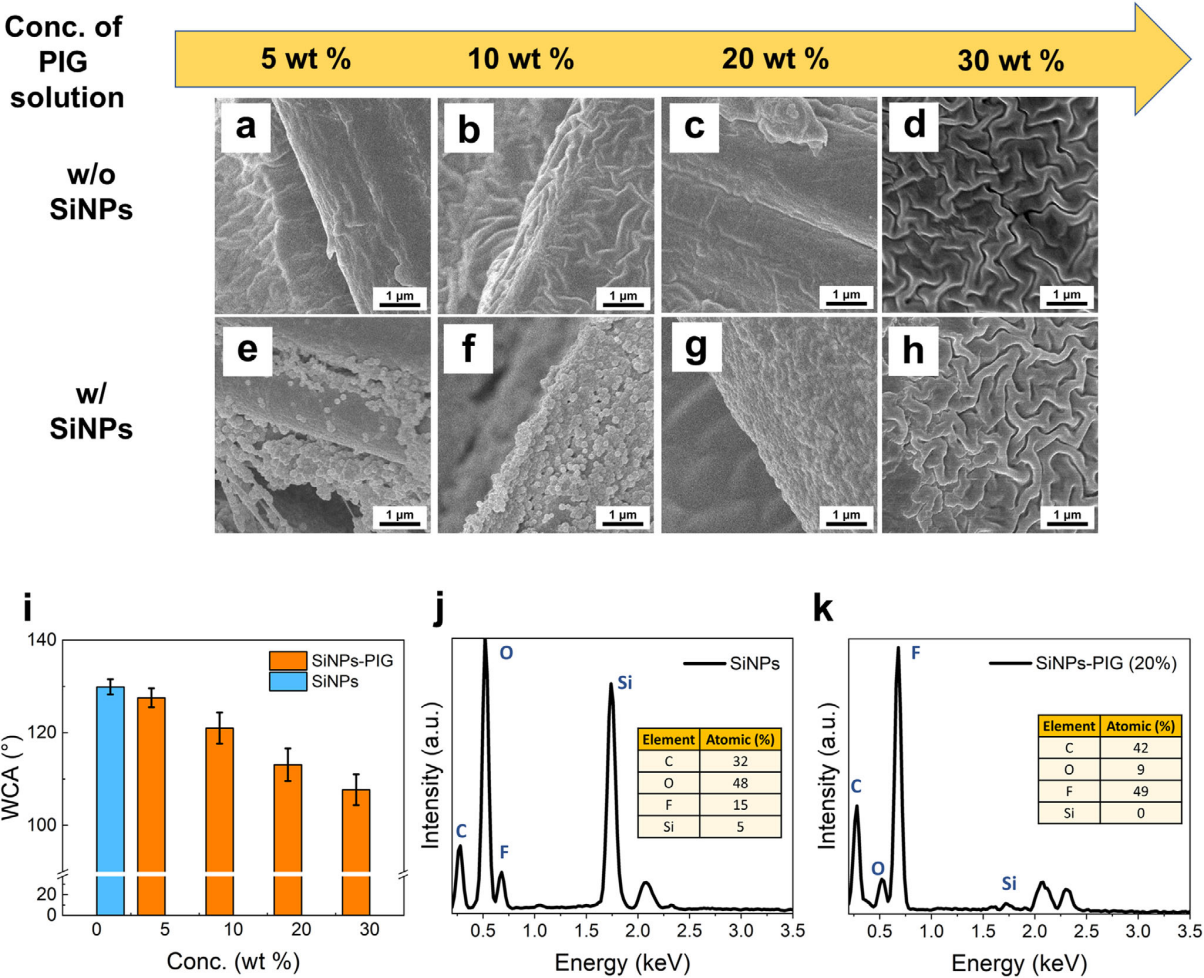
a–h) SEM images of the fabrics coated without and with SiNPs and coated with different concentrations of the PIG solutions. i) WCA of the fabrics coated without and with SiNPs and coated with different concentrations of the PIG solutions. j,k) EDS spectra of the fabrics coated with only SiNPs and coated with SiNPs and 20 wt.% of the PIG solutions.

In this study, SiNPs play a crucial role in enhancing the durability of the fabric coatings. However, as shown in Figure S6a (Supporting Information), when the PIG concentration is too low, the SiNPs remain exposed and disrupt the contact between the PIG layers, impairing the self‐healing capability. In contrast, when the PIG concentration is high (Figure S6b, Supporting Information), the SiNPs are fully covered by PIG layers, good contact between the PIG layers is re‐established and the self‐healing behavior is effectively restored. To evaluate how different coating conditions influence fabric morphology, SEM images of various samples are shown in **Figure** **3**a–h. Figure 3a–d depict fabrics coated with PIG only (without SiNPs), while Figure 3e–h shows fabrics coated with both SiNPs and PIG. At a low PIG concentration (5 wt.%), the SiNPs do not adhere effectively, resulting in significant detachment during fabrication and leaving the surface sparsely populated with SiNPs (Figure 3e). Increasing the PIG concentration to 10 wt.% improves SiNP adhesion to the fabric (Figure 3f). At higher concentrations (20 and 30 wt.%), the PIG forms a thicker coating that fully encapsulates the SiNPs, producing no notable morphological difference between samples with and without SiNPs (Figure 3c,d,g,h). Notably, at 30 wt.% PIG, the pores in the fabric structure are filled, leading to diminished breathability. These observations suggest that a 20 wt.% PIG solution provides an optimal balance, offering effective SiNPs adhesion while maintaining the fabric's structural integrity and breathability.

The surface wettability of the fabrics is assessed by water contact angle (WCA) measurements, as shown in Figure 3i. Uncoated fabrics are highly hydrophilic, with water droplets immediately soaking into the surface. Fabrics coated with SiNPs alone exhibit WCAs of ≈130°, indicating strong hydrophobicity due to the fluorinated SiNP surface. When both SiNPs and PIG are applied, the WCA decreases gradually with increasing PIG concentration, reaching ≈110°, a value close to that of the pure PIG coating.

To further investigate the surface composition, energy‐dispersive spectroscopy (EDS) and elemental mapping are conducted on fabrics coated with different combinations of SiNPs and PIG. For samples coated with SiNPs alone (Figure 3j; Figure S7, Supporting Information), the atomic percentages of silicon (Si) and fluorine (F) are measured at 5% and 15%, respectively. When coated with both SiNPs and 20 wt.% PIG, the Si signal disappears while the F signal increases to 49% (Figure 3k; Figure S8, Supporting Information), suggesting that the PIG fully covers the SiNPs. In contrast, samples coated with SiNPs and only 5 wt% PIG retain a detectable Si signal (4%) and show a more modest increase in F content (31%) (Figure S9a,b, Supporting Information), indicating that many SiNPs remain partially exposed. These findings reinforce the conclusion that a 20 wt.% PIG concentration achieves complete coverage of SiNPs, whereas lower concentrations do not. The elemental composition and distribution are further validated by electron probe microanalysis (EPMA), as shown in Figure S10 (Supporting Information).

**Figure 4:**
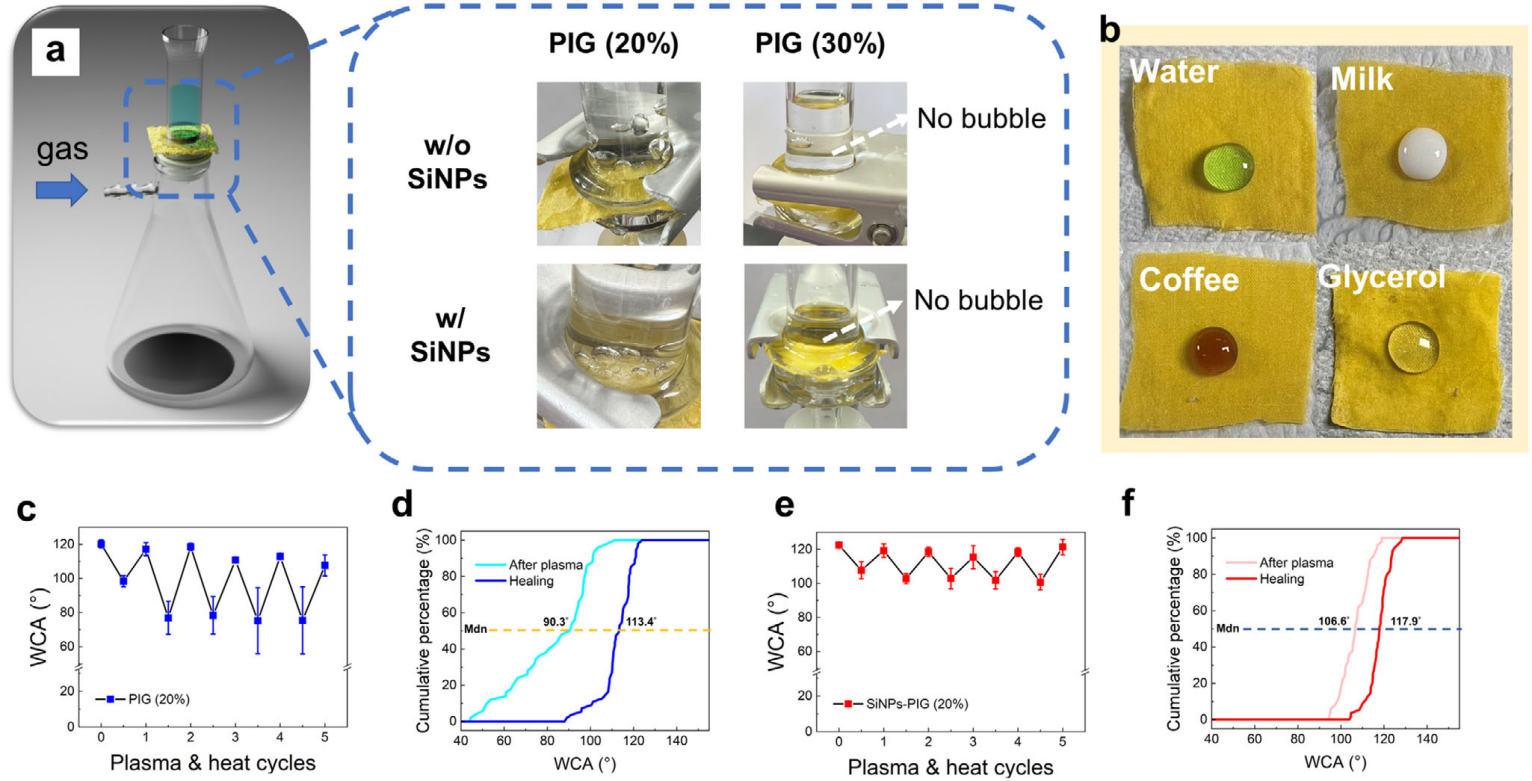
a) Schematic illustration and photos of the breathable tests for different fabrics. b) Photos of drops of water, milk, coffee, and glycerol on the fabrics coated with SiNPs and 20 wt.% of the PIG solutions. c,e) WCAs of the fabrics coated with 20 wt.% of the PIG solutions after plasma & heating cycle treatments: (c) without SiNPs and (e) with SiNPs. Cumulative percentage plots of the WCAs of the fabrics coated with 20 wt.% of the PIG solutions after plasma & heating cycle treatments: d) without SiNPs and f) with SiNPs.

The breathability of the coated fabrics is an important factor for their practical applications. If the fabrics lose breathability, their usability is significantly reduced. The breathability test setup and results are presented in **Figure** **4**a. In this test, the fabric samples are placed over the neck of a filtering flask. Water is poured on top of the fabrics, and air is introduced through the port of the flask. The formation of bubbles in the water indicates that air can pass through the fabric, confirming breathability. For fabrics coated with 30 wt.% PIG (with or without SiNPs), no bubbles are observed, suggesting a loss of breathability due to blockage of fabric pores. In contrast, fabrics coated with 20 wt.% PIG maintain breathability, indicating that the coating remains porous enough to allow gas flow, making them a more suitable choice.

In addition to breathability, the coated fabrics exhibit excellent liquid‐repellent performance, as shown in Figure 4b. Photographs demonstrate that water, milk, coffee, and glycerol droplets do not penetrate the surface of fabrics coated with 20 wt% SiNPs–PIG, indicating broad‐spectrum liquid repellency.

To more thoroughly evaluate the liquid repellency and durability of the coated fabric, a series of tests on SiNPs‐PIG (20%) are also conducted (Figures S11–S13, Supporting Information). Figure S11 (Supporting Information) is the water droplet retention test, by which the water droplet is left on the SiNPs‐PIG (20%) for 80 min. The water droplet gradually shrinks due to the evaporation of the water, rather than penetrating through the coated fabric, indicating strong water repellency. The result of prolonged water immersion test is shown in Figure S12 (Supporting Information). The SiNPs‐PIG (20%) is immersed in water for 72 h. The WCAs remain nearly unchanged, demonstrating that the coating layer keeps its water repellency in water for a long time. To test the durability of water repellency, mechanical agitation test is tested, by which the SiNPs‐PIG (20%) is stirred in water at 400 rpm using a 2.5 cm radius magnetic stirrer. As shown in Figure S13 (Supporting Information), the WCAs of SiNPs‐PIG (20%) remain above 112° even after continuous stirring, indicating good mechanical durability; the slight decrease of WCAs from 117 to 112° suggests minor surface abrasion but no serious damage.

For the porous and textured fabrics, the results of WCAs can be influenced by the surface morphology and may not fully reflect water repellency. However, the results of WCAs can reflect their roles as qualitative indicators of water repellency trends. To further support the water repellency property, the water absorption tests are conducted. As shown in Figure S14 (Supporting Information), the SiNPs‐PIG (20%) shows significantly lower water absorption than uncoated fabric, further supporting the result of water repellency.

The surface self‐repairing capability of the fabrics is attributed to the inherent self‐healing property of the PIG. Because the *T*_g_ of PIG is −29 °C,^[^ ^24^ ^]^ heating the sample can accelerate polymer mobility and improve healing efficiency. Incorporation of SiNPs further enhances the coating's durability of hydrophobicity, as the fluorinated groups on the SiNP surface improve interfacial adhesion between the PIG and the fabric. To assess this durability of hydrophobicity, water contact angles (WCAs) are measured over multiple O₂ plasma and heating cycles. Initially, O₂ plasma treatment renders the coated fabrics hydrophilic, lowering the WCA. Subsequent heating activates the surface self‐repairing function, partially or fully restoring the hydrophobicity. The WCA variations are shown in Figure 4c,e. For fabrics coated with 20 wt% PIG only, WCAs decrease substantially after plasma treatment and exhibit only partial recovery after heating (Figure 4c). In contrast, fabrics coated with both SiNPs and PIG show smaller fluctuations and near‐complete recovery (Figure 4e), indicating superior durability of hydrophobicity.

A more detailed analysis is presented in Figure 4d,f, which show cumulative distributions of WCA values over 10 cycles. For the PIG‐only sample, the median WCA drops to 90.3° after plasma treatment and recovers to 113.4° after heating (Figure 4d). In comparison, the SiNPs‐PIG (20%) sample maintains a higher median WCA of 106.6° after plasma treatment and recovers to 117.9° after heating (Figure 4f). Moreover, the range between maximum and minimum WCAs is reduced from 66.7° (PIG‐only) to 24.5° (SiNPs‐PIG (20%)), further confirming the enhanced durability of hydrophobicity conferred by the SiNPs.

**Figure 5:**
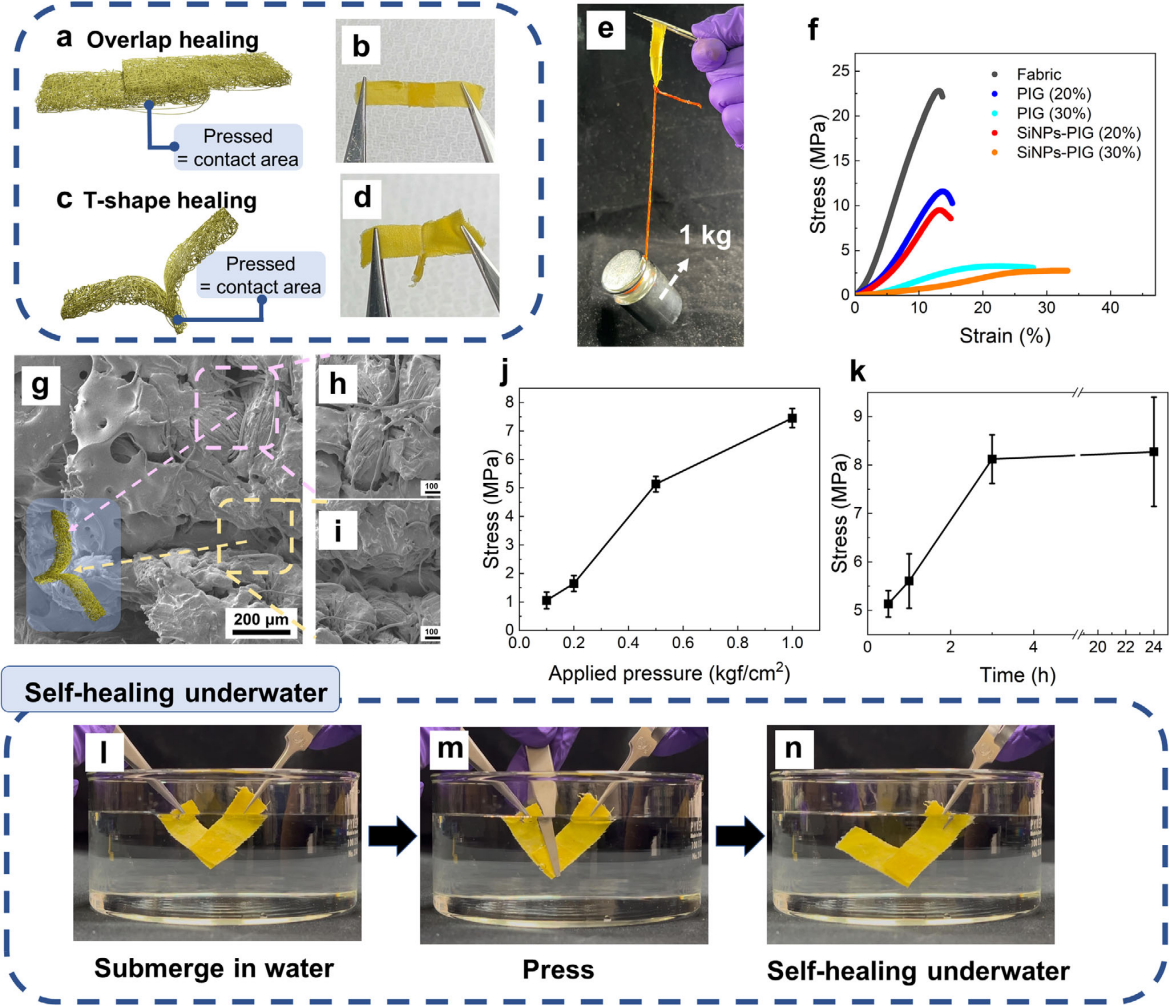
a) Illustration and b) photo of the overlapped self‐healed fabrics. c) Illustration and d) photo of the T‐shape self‐healed fabrics. e) Photo of the overlapped self‐healed fabrics loaded with a 1 kg weight. f) Stress–strain curves of the overlapped self‐healed fabrics in different coating conditions by applying 0.5 kgf cm^−2^ for 3 h. Top‐view SEM images of the self‐healed fabrics: g) the self‐healed boundary of the T‐shape self‐healed fabrics, h) the area without pressing, and i) the area after pressing. j) Plot of the maximum stress of the self‐healed fabrics by applying different pressures for 30 min. k) Plot of the maximum stress of the self‐healed fabrics by applying 0.5 kgf cm^−2^ for different times. l– n) Photos of the self‐healing process underwater of the fabrics.

The fabrics can perform notch self‐healing like PIG (Figure S15, Supporting Information), however, the ultrathin and flexible nature of fabrics causes precise realignment of the notch difficult. The SEM image for notch self‐healing (Figure S16, Supporting Information) demonstrates that healing only occurs at physical contact regions, while non‐contacted regions remain unhealed. This result shows the difficulty in aligning fractured interfaces due to the fabric's deformability and confirms that healing only occurs at contact points and is not due to general adhesive behavior because no bonding occurs at the non‐contacted interfaces. Therefore, the self‐healing performance of the fabrics is evaluated by joining two fabric pieces with a contact area of 1 cm^2^ and applying a pressure of 0.5 kgf cm^−^^2^ for 3 h.^[^ ^24^, ^25^, ^26^, ^35^ ^]^ Two configurations are tested: overlapped and T‐shaped joints, as illustrated in **Figure** **5**a–d. The overlapped configuration shows higher self‐healing efficiency, while the T‐shaped configuration is better suited for visualizing the healing interface.^[^ ^24^ ^]^ Figure 5e demonstrates the mechanical robustness of a healed sample, which supports a load exceeding 1 kg over the 1 cm^2^ bonding area.

To quantitatively assess healing performance, tensile tests are conducted on healed samples with different PIG concentrations, as shown in the stress–strain curves (Figure 5f). The presence or absence of SiNPs does not significantly affect the tensile behavior. Among the tested concentrations (10, 20, and 30 wt%), the 10 wt.% PIG fails to form sufficient ion–dipole interactions and shows negligible healing strength. Although 30 wt.% PIG provides the highest maximum force, its increased thickness reduces the effective stress‐bearing capacity. The 20 wt.% PIG formulation delivers a balanced performance, combining mechanical strength, breathability, and self‐healing efficiency.

The morphology of the healed interfaces is further analyzed using SEM. A top‐view image of the healed region is shown in Figure 5g. The corresponding unpressed area is shown in Figure 5h, and the cross‐sectional image of the healed interface is presented in Figure 5i. These images confirm that the fabric's porous structure is preserved even after pressing. The influence of applied pressure and healing time on healing performance is shown in Figure 5j,k. Increased applied pressure enhances the maximum stress of the healed joint (Figure 5j). Likewise, longer healing times improve bonding strength, with a plateau reached after 3 h. Beyond this point, further healing up to 24 h does not significantly increase the maximum stress, which stabilizes ≈8 MPa (Figure 5k).

Finally, the underwater self‐healing capability of the coated fabrics is evaluated, as shown in Figure 5l,m and Video S2 (Supporting Information). In the first part of the video, uncoated fabrics fail to adhere under mechanical force. In contrast, the coated fabrics form strong bonds under light pressure and remain attached even under vigorous agitation. This underwater healing performance underscores the potential of the developed materials for applications in wet or challenging environments.

To further assess the durability of the self‐healing property, the tests for immersion in strong acid and base are conducted, as shown in Figure S17a–c (Supporting Information). After 1 h immersion in strong acid and base, although the WCAs of SiNPs‐PIG (20%) slightly reduce by harsh pH exposure, they can be healed by the surface self‐repairing property of the PIG. (Figure S17a,b, Supporting Information) Moreover, the strong acid/ base treatments also have little influence on the self‐healing property; the overlapped self‐healing properties are both similar to the original one (Figure S17, Supporting Information). These results confirm that our coating system retains chemical durability in both hydrophobicity and self‐healing property.

The above data are mainly based on rayon fabrics. To further demonstrate the applicability of the two‐step dip‐coating method, polyester fabrics are also tested, as demonstrated in Figure S18 (Supporting Information). The coated polyester fabrics also exhibit better durability of hydrophobicity and surface‐repairing property (Figure S18a,b, Supporting Information). Additionally, the self‐healing of the coated polyester fabrics is also presented, as shown in Figure S18c (Supporting Information). The results indicate that the dip‐coating procedure can be applied to fabrics other than rayon. The slight variations in healing behavior can be attributed to differences in fabric structure, such as thickness and porosity, which affect the penetration and distribution of the PIG layer.

**Figure 6:**
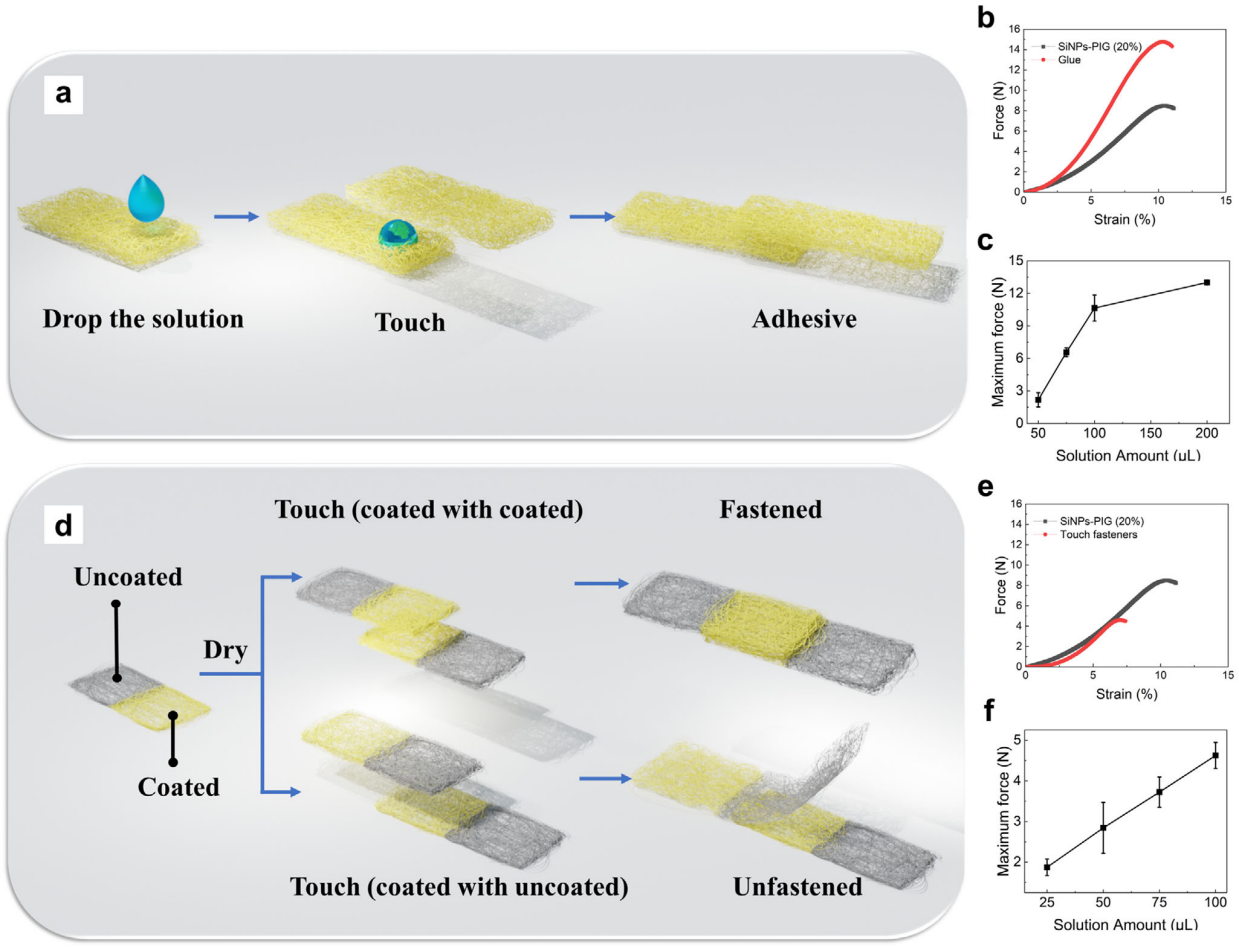
a) Schematic illustration of the adhesion process by using the PIG solution as glue. b) Plot of the force versus the strain of the self‐healed fabrics and the fabrics adhered by glue. c) Plot of the maximum force of the fabrics adhered by glue with different solution amounts. d) Schematic illustration of the fastening process by using the PIG solution as touch fastener. e) Plot of the force versus the strain of the self‐healed fabric and the fabric fastened by touch fastener for 1 cm^2^ area. f) Plot of the maximum force of the fabrics fastened by touch fastener with different coating solution amounts.

To explore the potential applications of the PIG solution beyond self‐healing fabrics, two functional strategies are investigated: use as an adhesive (glue) and as a touch fastener. The first approach evaluates the PIG solution as a glue between two fabric surfaces. As illustrated in **Figure** **6**a, a small volume of PIG solution is applied to a 1 cm^2^ area of one fabric sample, followed by the placement of another fabric piece on top. After allowing the assembly to sit for 1 min, the two fabrics bond together. The adhesive strength is then evaluated using a tensile testing machine (Figure 6b). With an identical bonding area of 1 cm^2^, the glued samples exhibit stronger adhesion than the self‐healed ones, with the maximum force increasing from 8 to 14 N. To further investigate the relationship between adhesive strength and the amount of applied solution, a series of tests with varying volumes of PIG are conducted, as shown in Figure 6c. Adhesive strength increases with solution volume up to ≈100 µL, beyond which further increases yield diminishing returns, likely due to excess surface energy and reduced cohesion.

In the second strategy, a touch fastener system is developed using selectively areal PIG coatings, as depicted in Figure 6d. In this design, designated regions of the fabric are coated with the PIG solution (yellow region), while the remaining areas remain uncoated (gray region). When a coated region contacts an uncoated one, no adhesion occurs. In contrast, strong adhesion is observed between two coated regions upon light pressure. This behavior mimics the function of a traditional touch fastener, but with additional benefits such as transparency, water repellency, and surface self‐repair. The mechanical performance of this selective adhesion is assessed by tensile testing (Figure 6e). After pressing the coated regions together at 0.5 kgf cm^−^^2^ for 30 min, the resulting adhesive strength is lower than that of the glue or fully self‐healed fabrics. The influence of the applied solution volume on the adhesion of the touch fastener is shown in Figure 6f, revealing a positive correlation between volume and bonding strength. To visually demonstrate the selectivity of the touch fastener, a video (Video S3, Supporting Information) is recorded. In the first section, uncoated regions are pressed together and fail to adhere, even under significant pressure. In contrast, when two PIG‐coated regions are brought into contact, they exhibit immediate and strong adhesion under slight pressure. This selective adhesion capability highlights the potential of the PIG system for advanced applications, such as customizable fastening and reusable adhesive materials.

## 3 Conclusion

In summary, this study presents a simple, scalable, and cost‐effective dip‐coating strategy to fabricate intrinsically self‐healing fabrics with excellent mechanical durability, breathability, and underwater healing capability. By integrating SiNPs and PIG coatings, the coated fabrics exhibit robust ion–dipole interaction‐based self‐healing properties at room temperature, even in aqueous environments. The incorporation of SiNPs not only enhances the coating durability of hydrophobicity and liquid repellency but also preserves the porous structure of the fabrics, enabling air permeability. Furthermore, the developed PIG solution demonstrates potential multifunctionality, serving as both an effective adhesive and a selective touch fastener. This work provides a promising platform for the development of smart textiles with advanced self‐healing, adhesive, and customizable fastening capabilities, offering significant potential for future applications in wearable electronics, protective clothing, and sustainable materials.

## 4 Experimental Section

### Materials

Tetraethyl orthosilicate (TEOS) was obtained from TCI. 1H,1H,2H,2H‐Perfluorodecyltriethoxysilane (FAS) and 1‐ethyl‐3‐methylimidazolium bis(trifluoromethylsulfonyl)imide (EMITFSI) were purchased from COMBI. Amorphous poly(vinylidene fluoride‐co‐hexafluoropropylene) (a‐PVDF‐HFP) was sourced from 3 M Dyneon. Crystalline poly(vinylidene fluoride‐co‐hexafluoropropylene) (c‐PVDF‐HFP) was acquired from Sigma–Aldrich. Rayon fabrics (thickness: 0.137 mm) and polyester fabrics (thickness: 0.264 mm) were purchased from Sing Way Fabrics.

### Preparation of the Silica Nanoparticles (SiNPs) Solutions

Initially, 4 mL ammonia was mixed with 50 mL ethanol and stirred until homogeneous. Subsequently, 4.5 mL TEOS was added and stirred at room temperature for 2 h. Finally, 0.25 mL FAS was introduced to the mixture, followed by stirring for an additional hour at room temperature, resulting in a SiNPs solution of ≈2.6 wt.%.

### Preparation of the Polymeric Ionic Gel (PIG) Solutions

Amorphous and crystalline PVDF‐HFP were mixed at a ratio of 7:3. The mixture was dissolved in acetone at various concentrations (5, 10, 20, and 30 wt.%). EMITFSI was subsequently added to each solution at a PVDF‐HFP:EMITFSI ratio of 7:3, forming the PIG solutions.

### Preparation of the Self‐Healing Fabrics

Self‐healing fabrics were fabricated using a two‐step dip‐coating method. Initially, fabric was immersed in the SiNPs solution for 1 min. After drying, the fabric was dipped into the PIG solution for another minute. The coated fabric was then dried at room temperature for 10 min to yield the final self‐healing fabric.

### Mechanical and Self‐Healing Test

Mechanical properties were evaluated using a tensile testing machine (Shimadzu EZ). Fabric samples (1 cm × 3 cm) were fixed onto plates (3 cm × 8 cm) containing a stretching hole (1 cm × 2 cm). For self‐healing assessments, fabric pieces (1 cm × 2 cm) were overlapped by 1 cm^2^ and pressed under a weight of 0.5 kg for 30 min. Tensile testing of healed samples was conducted at a strain rate of 5 mm min^−1^. Healing performance was assessed by comparing stress (MPa) and strain (%) values of coated and uncoated fabrics.

### Structure Analysis and Characterization

Sample morphologies were examined using scanning electron microscopy (SEM, JEOL JSM‐7401F) operated at 5 kV. Samples were vacuum‐dried for 5 h and coated with platinum (JEOL JFC‐1600 sputter coater, 20 mA, 50 s) prior to SEM analysis. Silica nanoparticle morphology was characterized via transmission electron microscopy (TEM, JEOL JEM‐2100) at 160 kV. Chemical analyses employed energy dispersive spectroscopy (EDS, Oxford EDS 7585), Fourier‐transform infrared spectroscopy (FTIR, PerkinElmer Spectrum One), X‐ray photoelectron spectroscopy (XPS, ULVAC‐PHI PHI Quantera II), and field‐emission electron probe microanalysis (FE‐EPMA, JEOL JXA‐8530F Plus). Particle size distributions of SiNPs were determined using dynamic light scattering (DLS) with a particle size analyzer (ELSZ‐2000S). Thermogravimetric analysis (TGA, TA55) was conducted from 50 to 700 °C for samples of 5 mg. Data processing utilized OriginPro 2024b software (OriginLab Corporation). Plasma and heating durability tests involved O_2_ plasma treatment (Advanced Research Technology Corporation, PPC‐1000, 145 V, 10 min), followed by heating at 60 °C for 1 h. Water contact angles (WCAs) were measured with a contact angle analyzer (First Ten Angstroms, FTA125).

## Conflict of Interest

The authors declare no conflict of interest.

## Supporting information



Supporting Information

Supplemental Video 1

Supplemental Video 2

Supplemental Video 3

## Data Availability

The data that support the findings of this study are available from the corresponding author upon reasonable request.
