# Supplementary material for: Achieving Intrinsically Self‐Healing Fabrics with Breathability, Surface Self‐Repairing, and Underwater Adhesion via Nanoparticle‐Polymer Gel Synergistic Coatings
Source: Small. 2025 Jun 18;21(32):2505120. doi: 10.1002/smll.202505120 (PMC12366270; doi:10.1002/smll.202505120)
Supplement: Supplementary file 1 — Supporting Information [file SMLL-21-2505120-s004.docx]

**Supporting Information**

Achieving Intrinsically Self-Healing Fabrics with Breathability, Surface Self-Repairing, and Underwater Adhesion via Nanoparticle-Polymer Gel Synergistic Coatings

Tsung-Hung Tsai,^1^ Lin-Ruei Lee,^1^ Yi-Chun Fan,^1^ Ruo-Yun Wei,^1^ Chia-Wei Chang,^1^ Huan-Wei Lin,^1^ Yu-Chun Lin,^1^ Tse-Yu Lo,^1^ Kesavan Manibalan,^1^ Ji Lin,^1^ Yen-Shen Hsu,^1^ Yu-Hsuan Tseng**,^1^** Hsun-Hao Hsu**,^1^** and Jiun-Tai Chen^1,2^*

^1^Department of Applied Chemistry, National Yang Ming Chiao Tung University, 300093 Hsinchu, Taiwan

^2^Center for Emergent Functional Matter Science, National Yang Ming Chiao Tung University, 300093 Hsinchu, Taiwan

^*^To whom correspondence should be addressed. E-mail: jtchen@nycu.edu.tw.


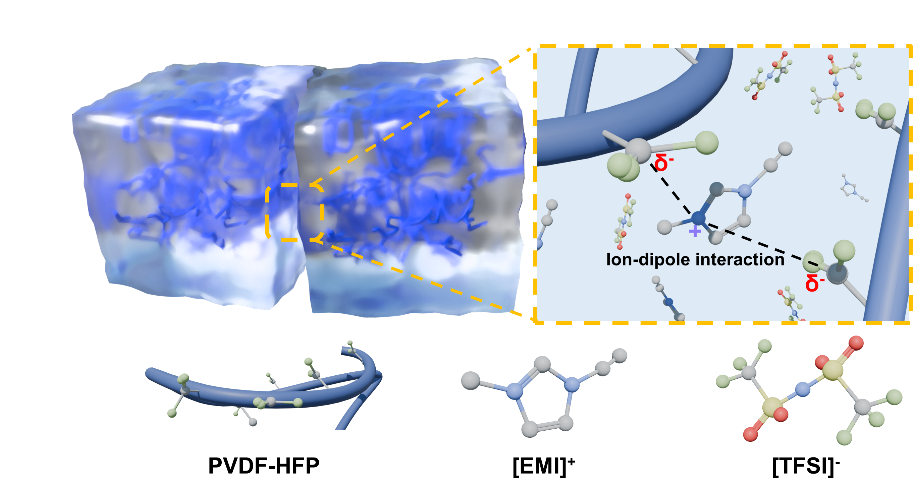


**Figure S1.** Schematic illustration of the self-healing mechanism.


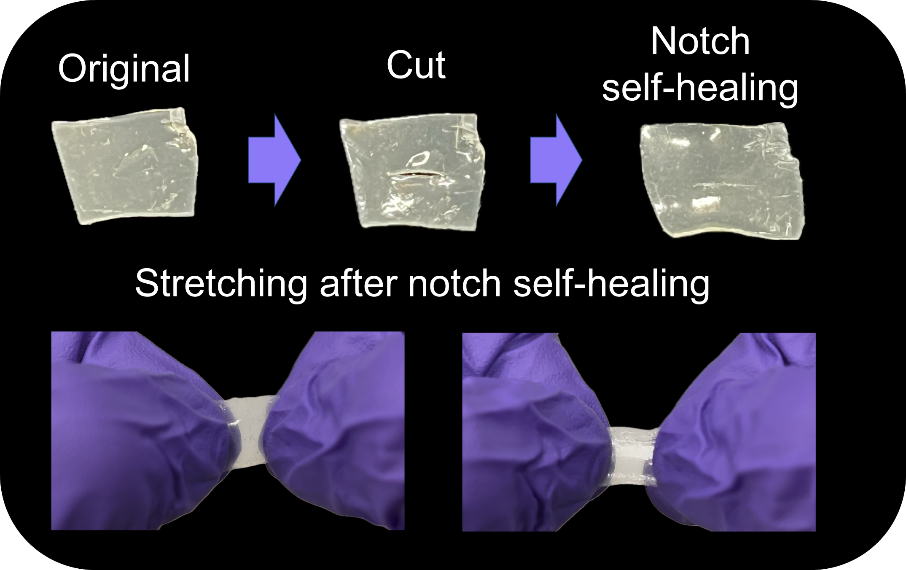


**Figure S2.** Photos of original and posthealed PIG.


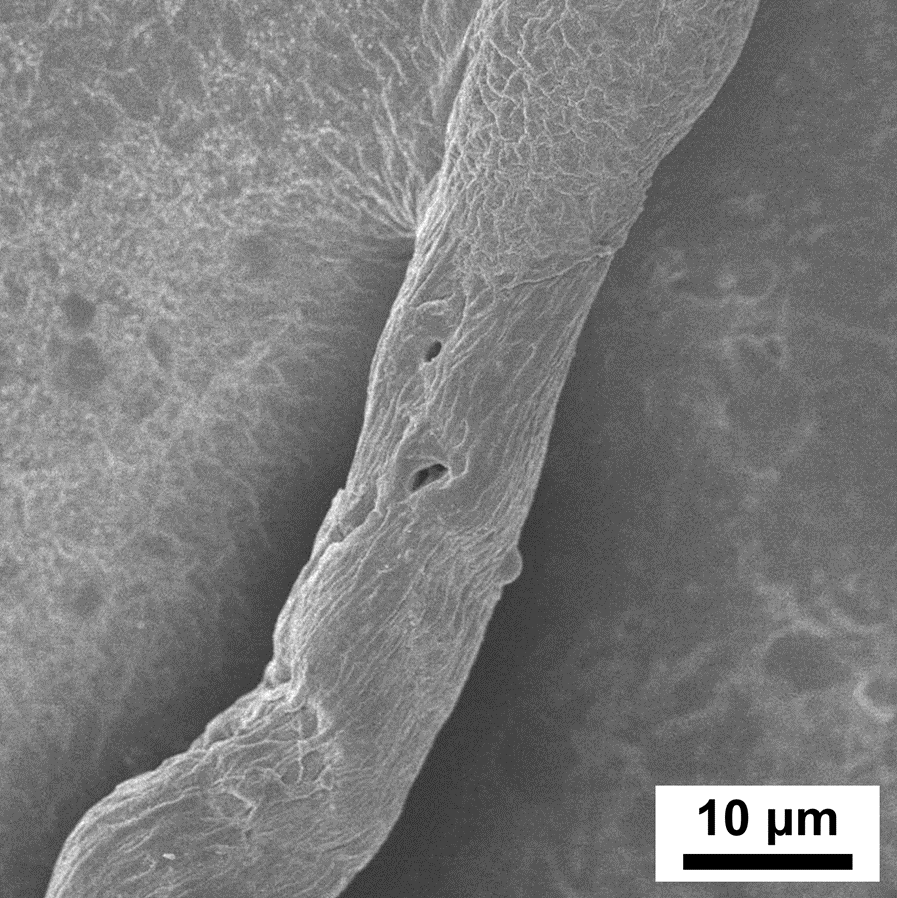


**Figure S3.** SEM image of the notched area of a PIG after healing.


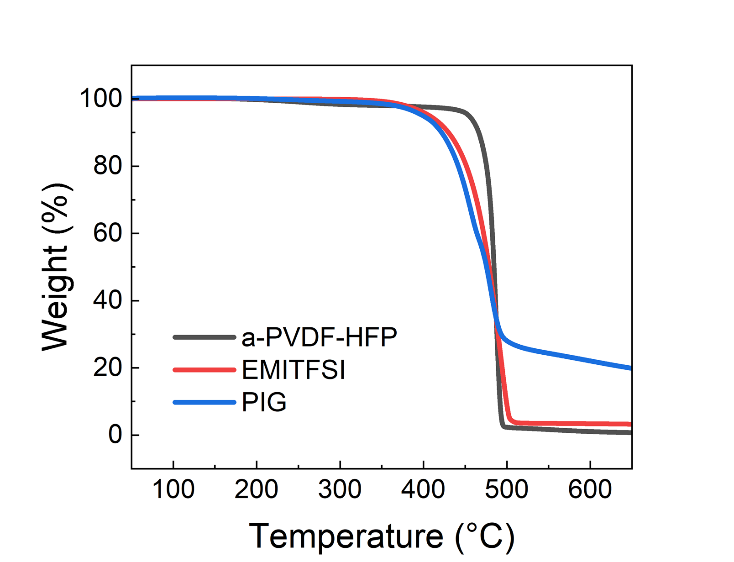


**Figure S4.** TGA curves of a-PVDF-HFP, EMITFSI, and PIG.


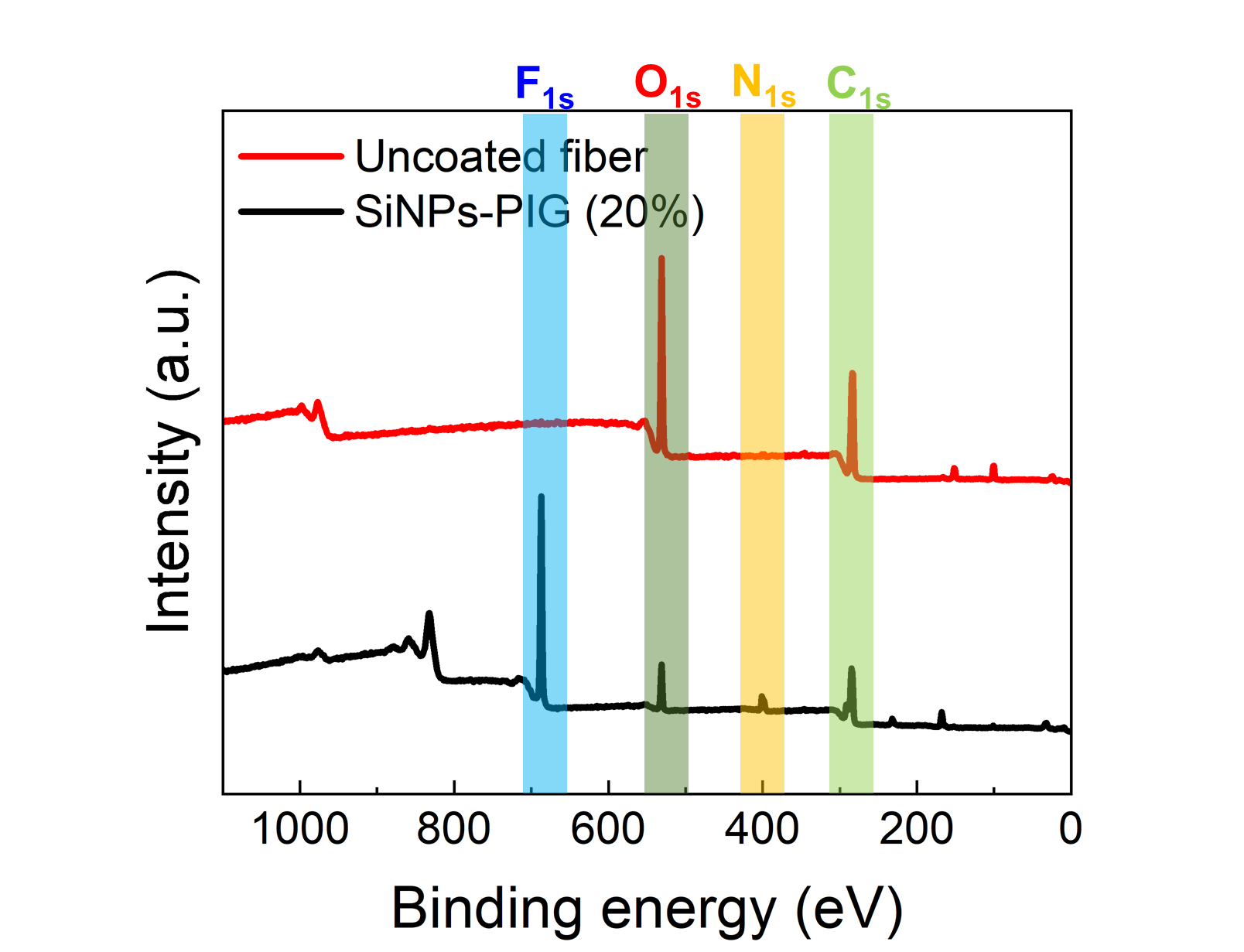


**Figure S5.** XPS whole spectra of the uncoated fabrics and fabrics coated with SiNPs and 20 wt % of the PIG solutions.


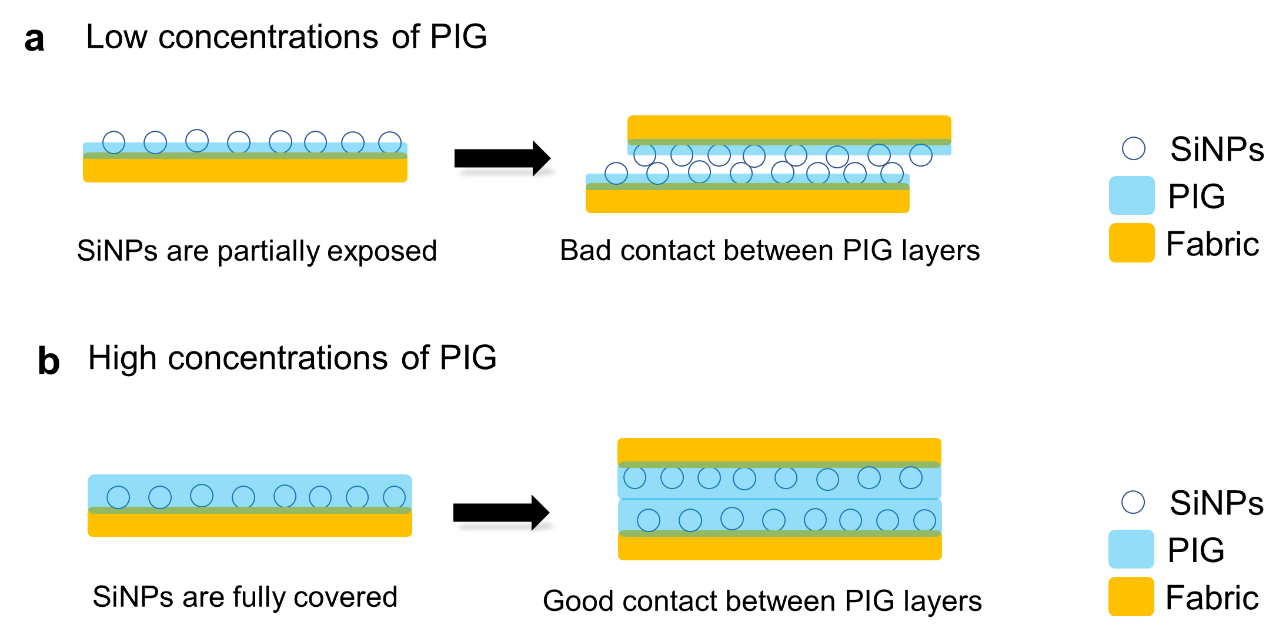


**Figure S6.** Schematic illustrations of the contact between PIG layers for (a) low concentrations and (b) high concentrations of PIG.

**
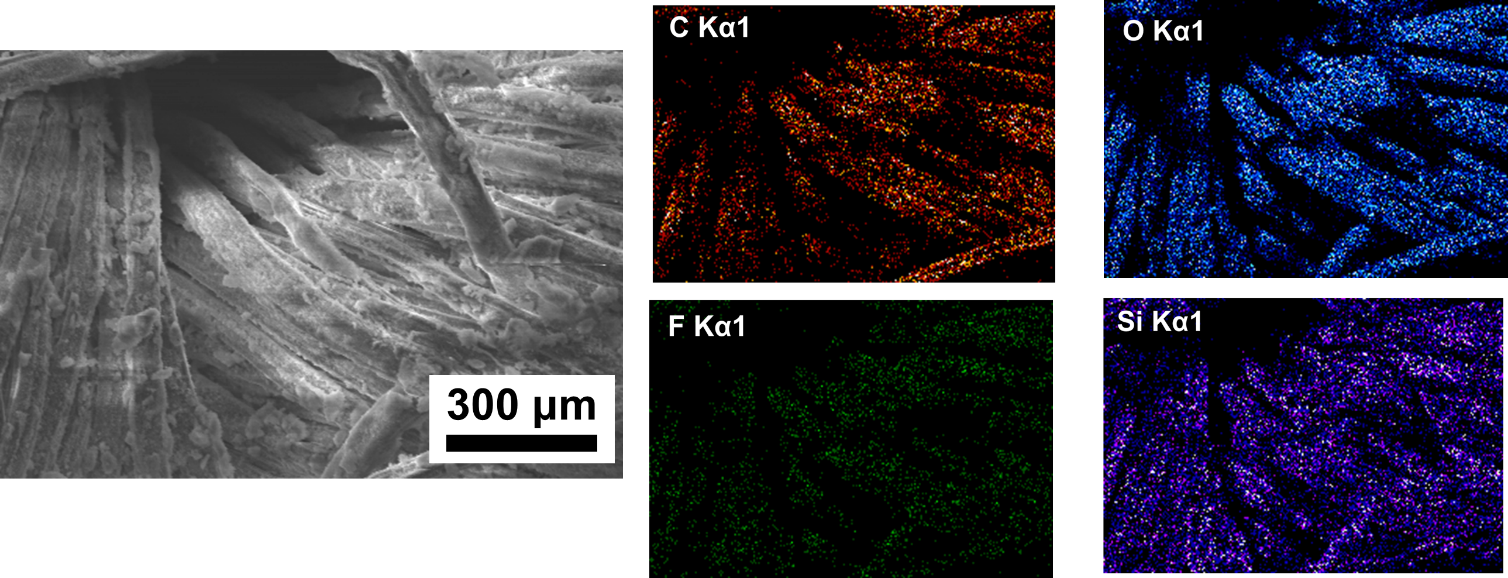
**

**Figure S7.** SEM image and corresponding EDS elemental mapping of the fabrics coated with pure SiNPs.


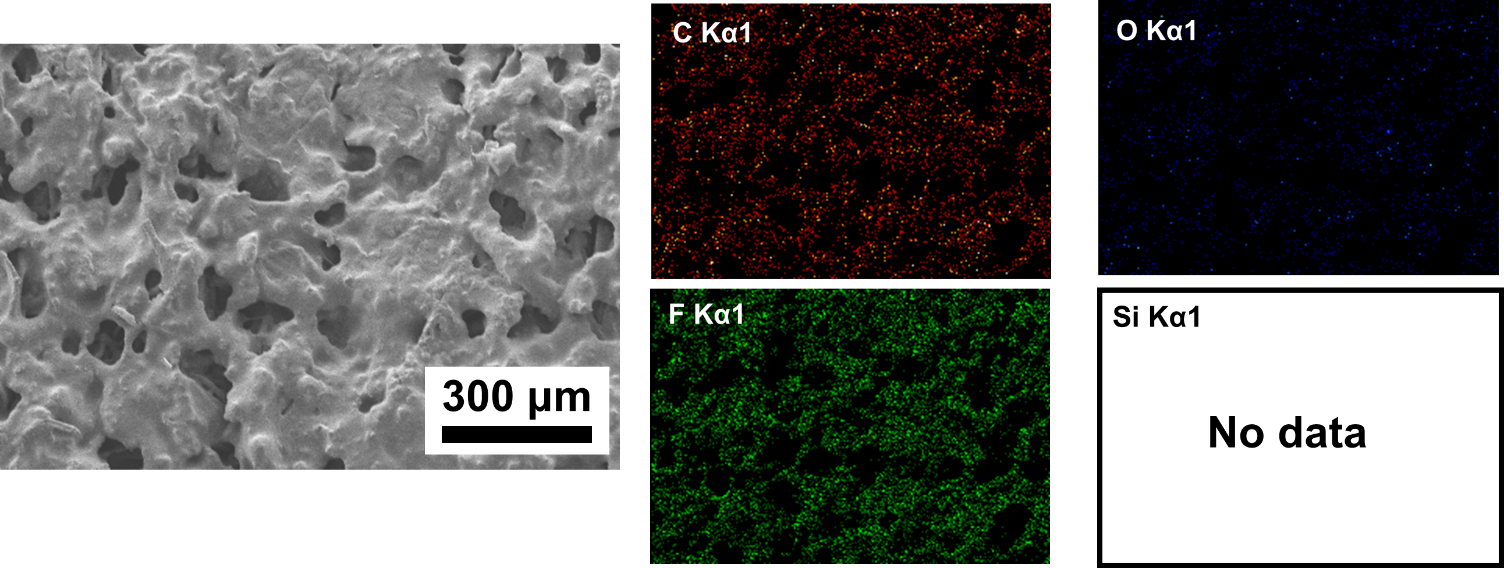


**Figure S8.** SEM image and corresponding EDS elemental mapping of the fabrics coated with SiNPs and 20 wt % of the PIG solutions.


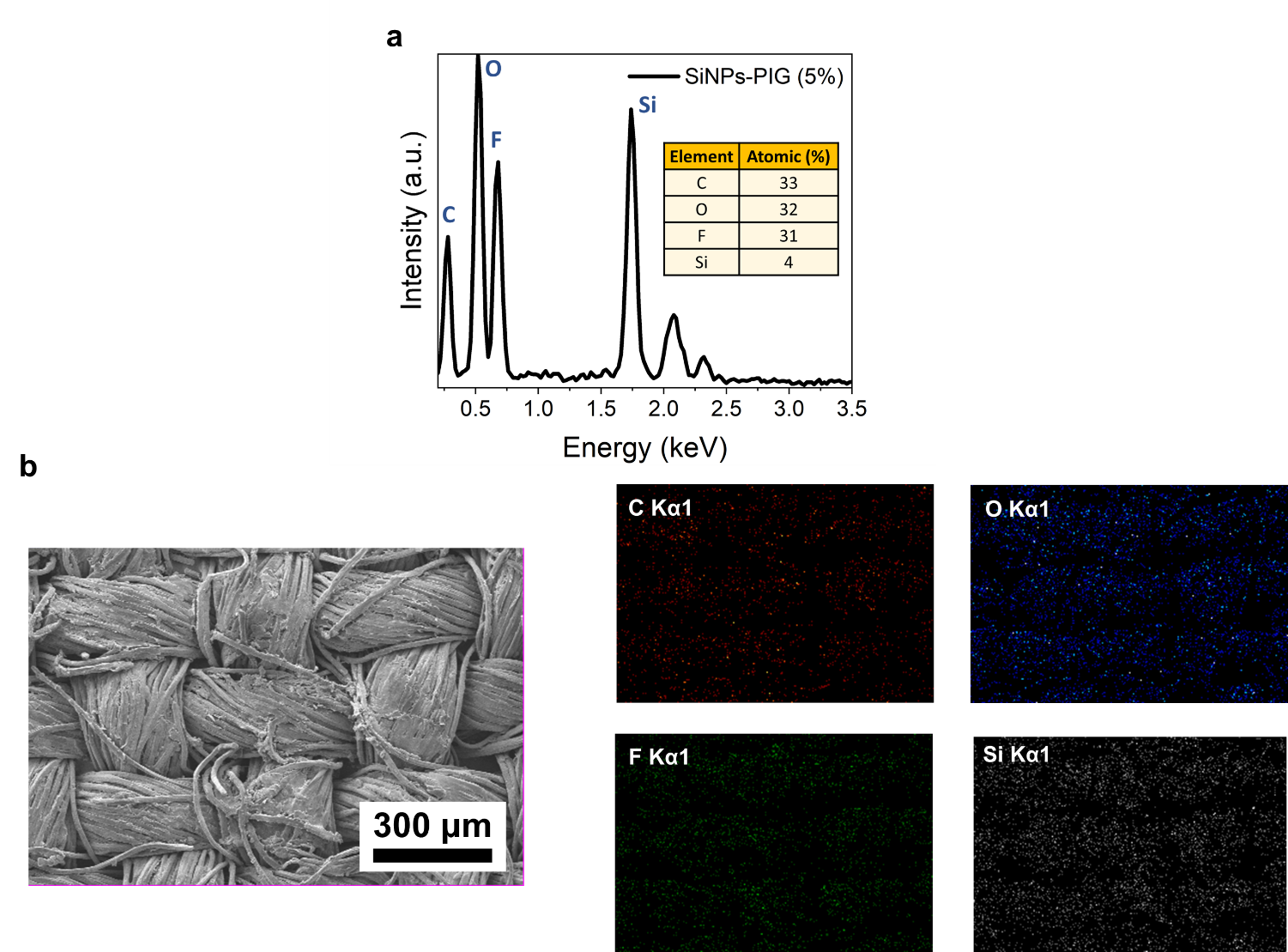


**Figure S9. (**a) EDS spectrum and (b) SEM image and corresponding EDS elemental mapping of the fabrics coated with SiNPs and 5 wt % of the PIG solutions.


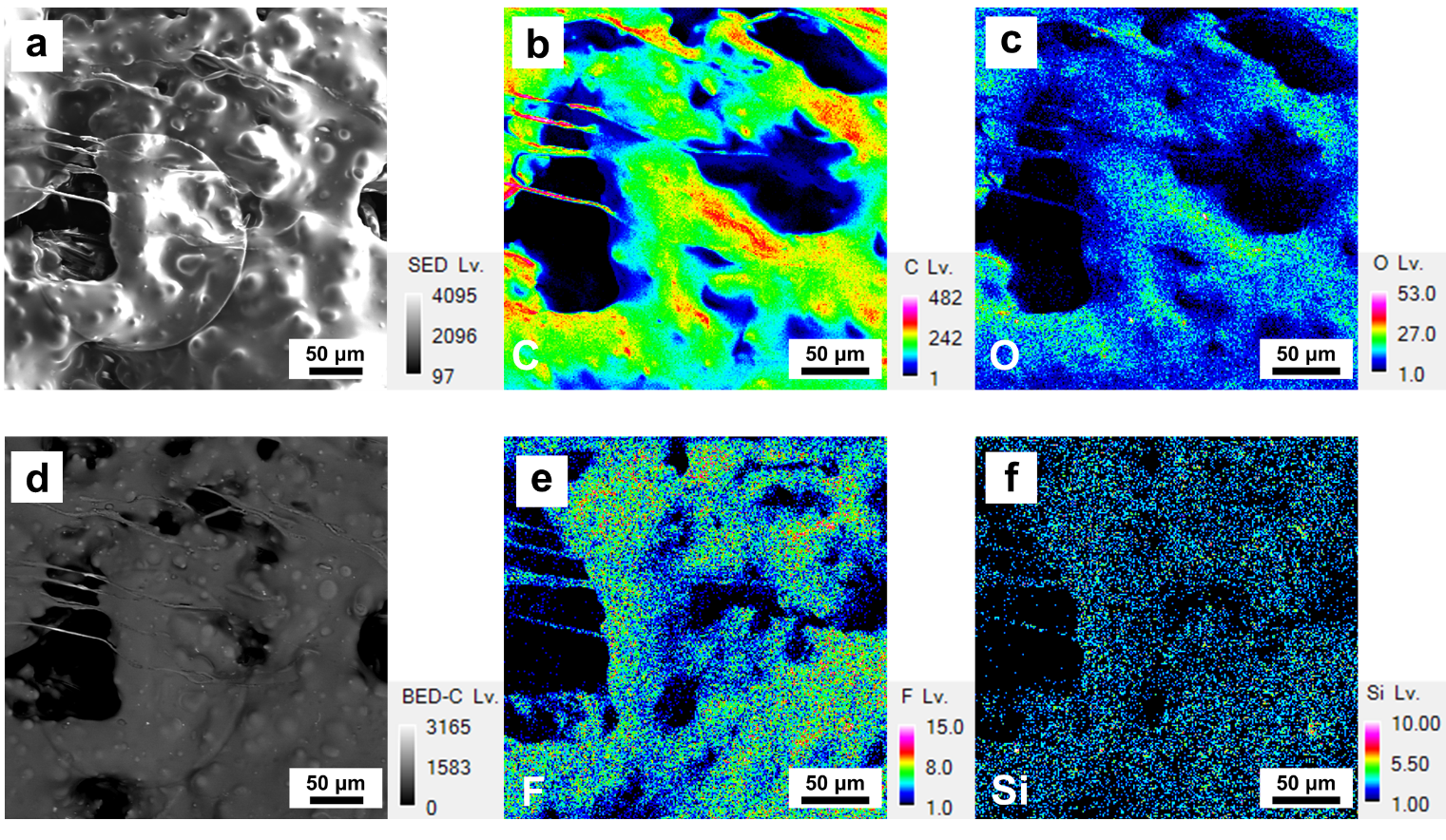


**Figure S10.** (a) Secondary electron image and (d) backscattered electron image of the fabrics coated with SiNPs and 20 wt % of the PIG solutions. (b, c, e, f) Corresponding elemental WDXS mapping of the fabrics coated with SiNPs and 20 wt % of the PIG solutions.


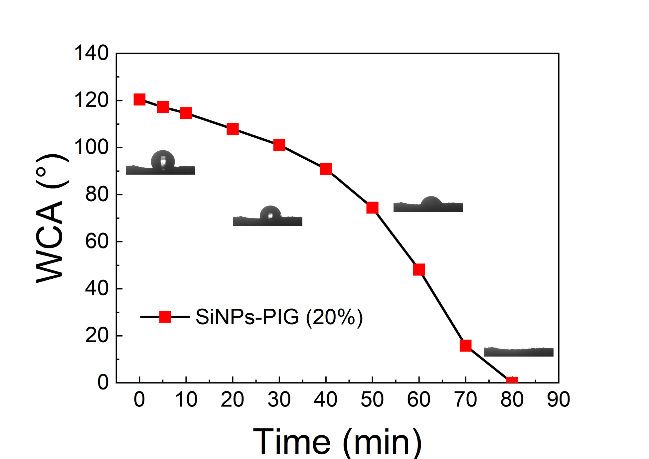


**Figure S11.** Time-lapse observation of a 4 μL water droplet placed on SiNPs-PIG (20%) coated fabric for 80 min.


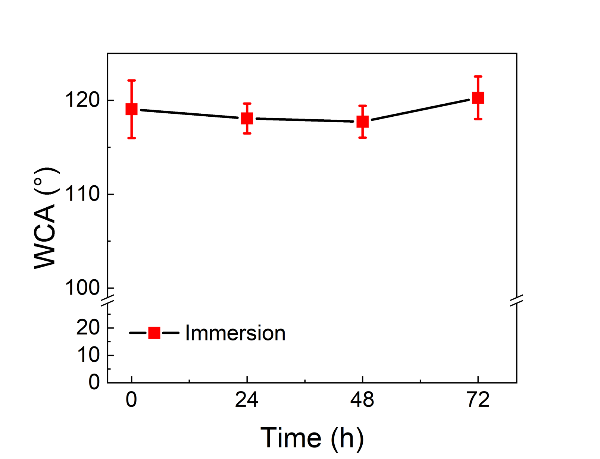


**Figure S12.** WCAs of SiNPs-PIG (20%) coated fabric after immersion in water for 72 h.


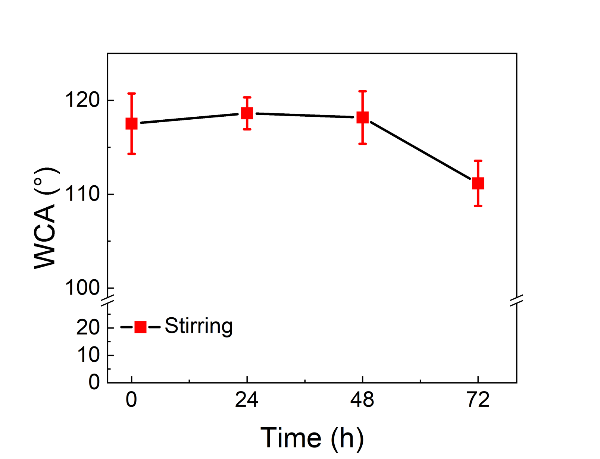


**Figure S13.** WCAs of SiNPs-PIG (20%) after mechanical agitation in water at 400 rpm using a 2.5 cm radius magnetic stirrer for different times.


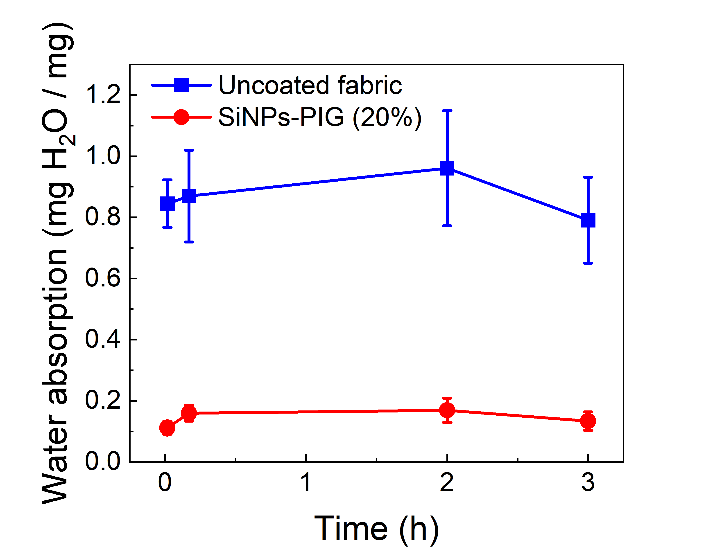


**Figure S14.** Water absorption of uncoated fabric and SiNPs-PIG (20%) after 3 h immersion.


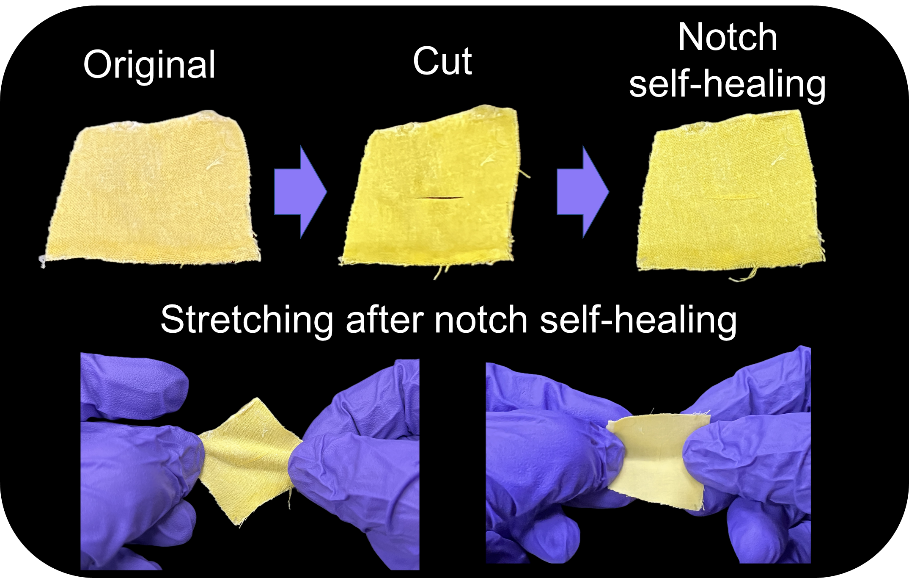


**Figure S15.** Photos of original and posthealed fabrics.


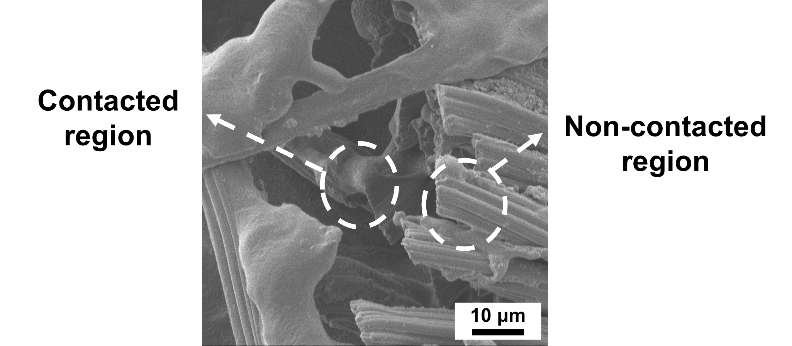


**Figure S16.** SEM image of the interface of the fabric after notch self-healing process.


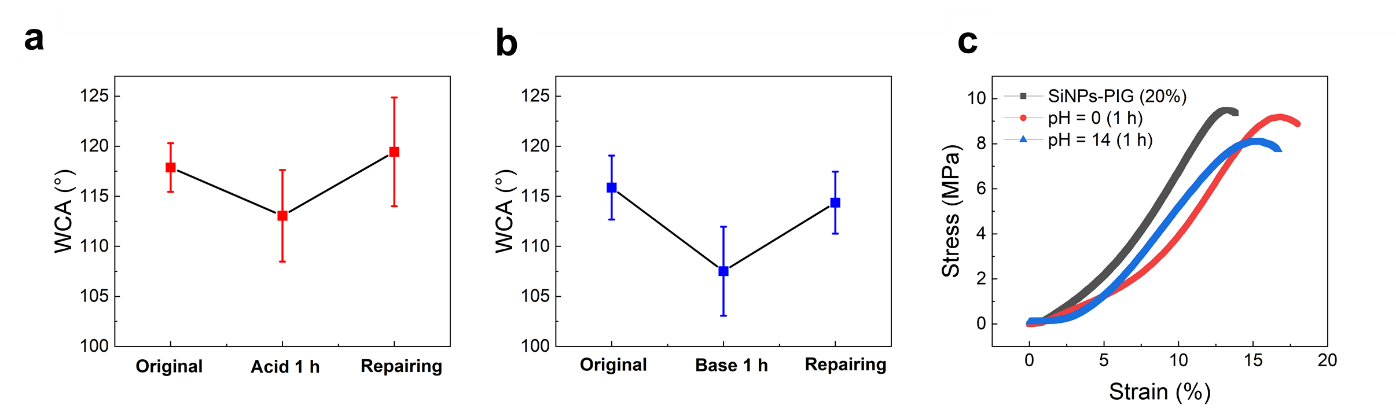


**Figure S17.** (a-b) WCAs of SiNPs-PIG (20%) in (a) 1 M HCl(aq) (pH = 0) and (b) 1M NaOH(aq) (pH = 14) for 1 h. (c) Overlapped self-healing after immersion in acid and base.


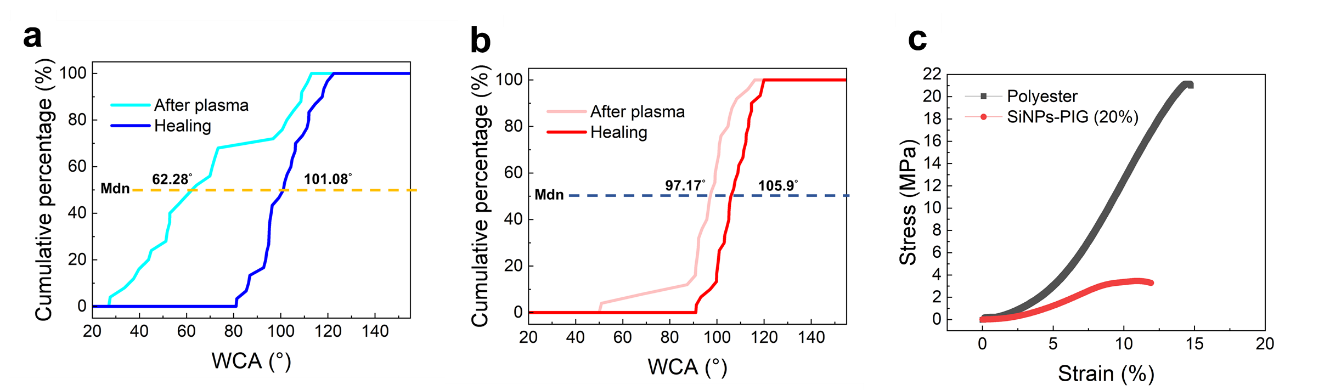


**Figure S18.** (a,b) Cumulative percentage plots of the WCAs of the polyester fabrics coated with 30 wt % of the PIG solutions after plasma & heating cycle treatments: (a) without SiNPs and (b) with SiNPs. (c) Stress-strain curves of the overlapped self-healed polyester fabrics.
